# Computational Analysis and Low-Scale Constitutive Expression of Laccases Synthetic Genes *GlLCC1* from *Ganoderma lucidum* and *POXA 1B* from *Pleurotus ostreatus* in *Pichia pastoris*

**DOI:** 10.1371/journal.pone.0116524

**Published:** 2015-01-22

**Authors:** Claudia M. Rivera-Hoyos, Edwin David Morales-Álvarez, Sergio Alejandro Poveda-Cuevas, Edwin Alfredo Reyes-Guzmán, Raúl A. Poutou-Piñales, Edgar Antonio Reyes-Montaño, Aura Marina Pedroza-Rodríguez, Refugio Rodríguez-Vázquez, Ángela M. Cardozo-Bernal

**Affiliations:** 1 Laboratorio de Biotecnología Molecular, Grupo de Biotecnología Ambiental e Industrial (GBAI), Departamento de Microbiología, Facultad de Ciencias, Pontificia Universidad Javeriana, Bogotá, D.C., Colombia; 2 Laboratorio de Microbiología Ambiental y Suelos, Grupo de Biotecnología Ambiental e Industrial (GBAI), Departamento de Microbiología, Facultad de Ciencias, Pontificia Universidad Javeriana, Bogotá, D.C., Colombia; 3 Departamento de Química y Grupo de Investigación en Genética, Biodiversidad y Manejo de Ecosistemas (GEBIOME), Facultad de Ciencias Exactas y Naturales, Universidad de Caldas, Manizales-Caldas, Colombia; 4 Escuela de Ciencias Biológicas, Facultad de Ciencias Básicas, Universidad Pedagógica y Tecnológica de Colombia (UPTC), Tunja-Boyacá, Colombia; 5 Departamento de Química, Facultad de Ciencias, Universidad Nacional de Colombia (UNAL), Bogotá, D.C., Colombia; 6 Departamento de Biotecnología y Bioingeniería, Centro de Investigaciones y de Estudios Avanzados del Instituto Politécnico Nacional (IPN), México, D.F., México; National Centre for Cell Science, INDIA

## Abstract

Lacasses are multicopper oxidases that can catalyze aromatic and non-aromatic compounds concomitantly with reduction of molecular oxygen to water. Fungal laccases have generated a growing interest due to their biotechnological potential applications, such as lignocellulosic material delignification, biopulping and biobleaching, wastewater treatment, and transformation of toxic organic pollutants. In this work we selected fungal genes encoding for laccase enzymes *GlLCC1* in *Ganoderma lucidum* and *POXA 1B* in *Pleurotus ostreatus*. These genes were optimized for codon use, GC content, and regions generating secondary structures. Laccase proposed computational models, and their interaction with ABTS [2, 2′-azino-bis(3-ethylbenzothiazoline-6-sulphonic acid)] substrate was evaluated by molecular docking. Synthetic genes were cloned under the control of *Pichia pastoris* glyceraldehyde-3-phosphate dehydrogenase (GAP) constitutive promoter. *P. pastoris* X-33 was transformed with pGAPZαA-*LaccGluc-Stop* and pGAPZαA-*LaccPost-Stop* constructs. Optimization reduced GC content by 47 and 49% for *LaccGluc-Stop* and *LaccPost-Stop* genes, respectively. A codon adaptation index of 0.84 was obtained for both genes. 3D structure analysis using SuperPose revealed LaccGluc-Stop is similar to the laccase crystallographic structure 1GYC of *Trametes versicolor*. Interaction analysis of the 3D models validated through ABTS, demonstrated higher substrate affinity for LaccPost-Stop, in agreement with our experimental results with enzymatic activities of 451.08 ± 6.46 UL^-1^ compared to activities of 0.13 ± 0.028 UL^-1^ for LaccGluc-Stop. This study demonstrated that *G. lucidum GlLCC1* and *P. ostreatus POXA 1B* gene optimization resulted in constitutive gene expression under GAP promoter and α-factor leader in *P. pastoris*. These are important findings in light of recombinant enzyme expression system utility for environmentally friendly designed expression systems, because of the wide range of substrates that laccases can transform. This contributes to a great gamut of products in diverse settings: industry, clinical and chemical use, and environmental applications.

## Introduction

At present dye use exceeds over 7 x 10^5^ tons/year in the paper, textile, and leather tanning industries [1]. Synthetic dyes are chemically classified as anthraquinones, sulfur derivatives, azo and phtalocyanine derivatives, as well as indigoid and triphenylmethyl compounds [2]. An aromatic ring in its structure characterizes all of these compounds. In addition to the problem the dye poses, most of these compounds are toxic, carcinogenic, and highly recalcitrant.

Furthermore, conventional biological treatments to degrade dye-contaminated waters are not effective. Thus, non-conventional strategies are required to eliminate them. Among them are physical and chemical techniques that include organic or inorganic matrix adsorption, and discoloration through advance oxidation processes [3–5].

Some lignolytic microorganisms, or also known as white rot fungi, among them, *Pleurotus ostreatus* and *Ganoderma lucidum* are capable of degrading a wide variety of aromatic structure contaminated compounds that are similar to lignin and its derivatives. Generally, Basidiomycetes are considered the most efficient lignin degraders, due to their principally laccase enzymatic machinery (EC 1.10.3.2), lignin peroxidases (EC 1.11.10.14), and manganese peroxidases (EC 1.11.1.13). This enzymatic endowment results very attractive for biotechnological applications, because it can catalyze toxic compound oxidation at the same time molecular oxygen is reduced to water. In addition, in comparison to other peroxidases, they are highly stable, facilitating their immobilized use [6–9]. Lignolytic enzyme production economic disadvantages include long production periods and low yields. However, recent studies have demonstrated direct laccase employment resulting in fast and significant substrate degradation [10].

In contrast, yeast display rapid growth that can be readily genetically manipulated. In addition, they carry out specific post-translational modifications such as proteolytic processing, disulfide-bond formation, and glycosylation. Thus, they are widely employed as a recombinant enzyme expression system. Additionally, yeast culture produces high yields, is inexpensive, and demands little in terms of time and culture media [11]. All these attributes represent an advantage for enzyme production at higher scales [9].

Generally, to increase yeast recombinant enzyme production, various strategies have been used, such as chromosomal multiple DNA gene copy integration. In addition, strong promoters have been employed. Furthermore, efficient signaling peptides in expression vectors have been selected to assure protein of interest over-expression by the host, and its secretion to the culture media. Hence, downstream processes are simplified; since the recombinant protein can be obtained from the crude extract simply by centrifuging the culture without any cell lysis [11–13].

Thus far, laccase fungal heterologous expression in yeast such as *Saccharomyces cerevisiae* [14], *Pichia pastoris* [15, 16]*, Pichia methanolica* [17]*, Yarrowia lipolytica* [18], and *Kluyveromyces lactis* [19] has been mainly performed by inducible promoters with encouraging results. Non the less, the methodology required to obtain high expression levels and enzyme biological activity is still controversial. Some researchers consider Ascomycetes a more suitable host, due to its genetic manipulation ease and scaling to industrial processes. However, higher redox-potential have been obtained from recombinant laccases in Basidomycetes, which are not as easy to manipulate genetically [11, 20, 21].

The capability to efficiently produce laccases in heterologous systems depends mainly on original DNA sequence modifications by genetic manipulation. As a case in point, enzyme secretion increase has been achieved by replacing the native signaling peptide sequence for increased secretion signals directly from the host [20, 22]. An additional factor that can influence recombinant protein expression is the chemical synthesis of the gene to be expressed. By using modified codons in a synonymous manner, translation can be facilitated. In addition, it diminishes host’s use of unusual codons. Bulter *et al.*, (2003) reported these modifications increased *Myceliophtora thermophila* laccase expression in *S. cerevisiae* [23]. Despite these reports it is still uncertain which can be considered the “ideal” host, the “most successful strategy” or the “most promising laccase”.

This work had several objectives, the first to optimize *GlLCC1* and *POXA 1B* sequences to assure that once synthesized they could be replicated, transcribed, and translated in *P. pastoris* as if they were its own. The second objective was to propose and computationally validate a 3D structure model for laccases GlLCC1 and POXA 1B or Lacc6 from *G. lucidum* and *P. ostreatus,* respectively. The third objective was to analyze by molecular docking the interaction between ABTS substrate, commonly used for biological enzyme activity quantification, and the laccases previously mentioned. Last, to validate our computational model in *P. pastoris* by heterologous constitutive expression of both synthetic genes *GlLCC1* and *POXA 1B* sequences.

Advancement in processes associated with recombinant laccase higher yield production by using the expression system in *P. pastoris* is very valuable.Our results contribute to the understanding of this process. Furthermore, we shed light on events related to reaction mechanisms of laccases. In addition, results from this study help elucidate interaction between these two laccases and different substrates on which they can exert their action.

## Results

### *LaccGluc-Stop* and *LaccPost-Stop* gene design and optimization

**Figure 1:**
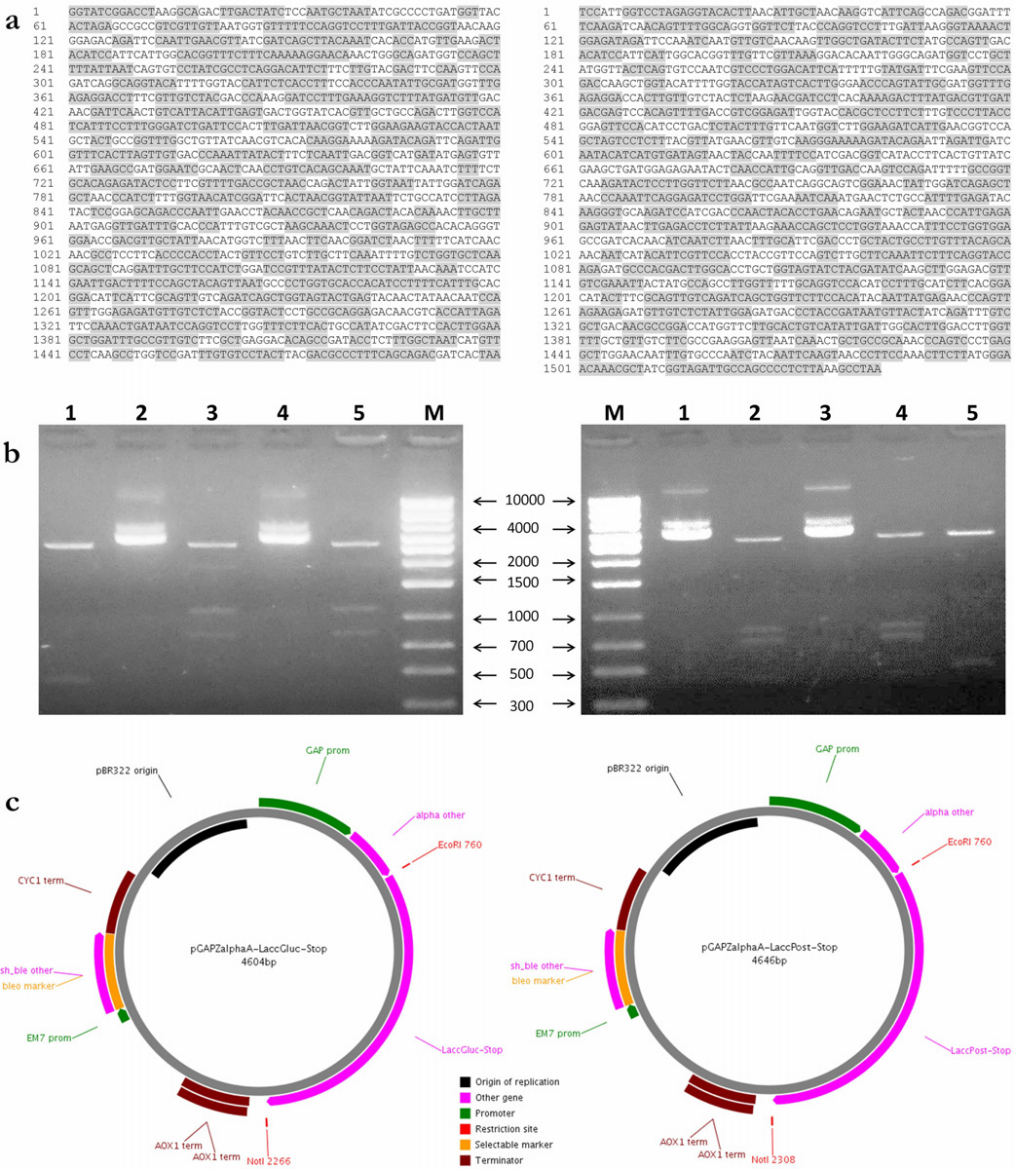
Analysis of cloning process. **a**. *LaccGluc-Stop* and *LaccPost-Stop* optimized sequences to be expressed in *P. pastoris.* **b**. 1% TAE gel electrophoresis for expression vector analysis. Left gel pGAPZαA-*LaccGluc-Stop.* pGAPZαA-*LaccGluc-Stop* (lanes 1 and 3), pGAPZαA-*LaccGluc-Stop* digested with *Eco*RI and *Bam*HI (lanes 2 and 4), pGAPZαA digested with *Eco*RI and *Bam*HI (lane 5). To the right: pGAPZαA-*LaccPost-Stop*. pGAPZαA digested with *Eco*RI and *Bam*HI (lane 1), pGAPZαA-*LaccPost-Stop* (lanes 2 and 4), pGAPZαA-*LaccPost-Stop* digested with *Eco*RI, *Bam*HI and *Hind*III (lanes 3 and 5) with the synthetic sequences *GlLCC1* and *POXA 1B.* M: Molecular weight size marker 1 kb (Axygen, Biosciences Tewksbury, MA USA). **c**. pGAPZαA-*LaccGluc-Stop* and pGAPZαA-*LaccPost-Stop* constructs transformed in *P. pastoris* X-33.

In this study we evidenced fungi *G. lucidum* and *P. ostreatus* codon use differed substantially from *P. pastoris*. *GlLCC1* and *POXA 1B* gene sequences included some codons that are utilized at low frequency by *P. pastoris*, with codon adaptation index (CAI) of 0.58 and 0.61, respectively. After optimization CAI values increased to 0.84 for both sequences. Likewise, we optimized GC content. For *LaccGluc-Stop* final sequence was reduced from 57.44% to 43.81%, for *LaccPost-Stop* decrease was from 54.83% to 43.88%. For both cases GC percentage was between 47–49%, values within the range of *P. pastoris.* In addition, we optimized sequences involved in instabilities associated with mRNA and secondary structure formation [24]. Lastly, for each sequence we added restriction enzyme recognition sites for *Eco*RI and *Not*I at 5´ and 3´ ends respectively (Fig. 1).

### Structural analysis for models constructed by homology

**Figure 2:**
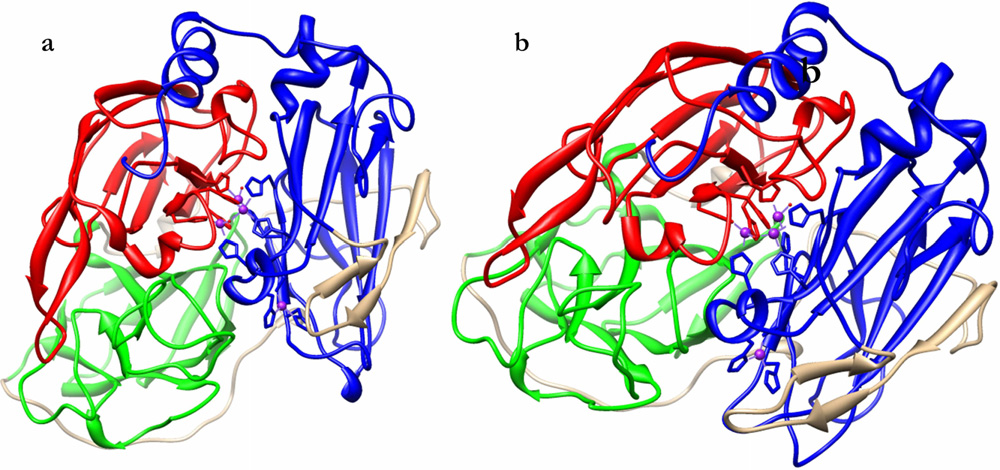
LaccGluc and LaccPost 3D structure. a. LaccGluc **b.** LaccPost. Domains: D1 (red), D2 (green) and D3 (blue). Copper ions can be observed as purple spheres and oxygen atoms as red spheres.

Preliminary pBLAST analysis, using PDB as database, showed that *T. versicolor* laccase has a high sequence identity with *G. lucidum* (LaccGluc) and *P. ostreatus* (LaccPost) laccases. Secondary structure prediction (PsiPred, DisoPred) showed identity also at a structural level. 120 different models for *G. lucidum* (LaccGluc) and *P. ostreatus* (LaccPost) laccase sequences were predicted by using Phyre. The highest grade obtained was for models constructed based on a *T. versicolor* crystallographic structure in Protein Data Bank (PDB) with access code 1GYC (1.9 Å) [25]. LaccGluc and LaccPost models presented 77% and 62% identities with respect to 1GYC, and 100% confidence values for both cases (Fig. 2).

To determine if copper ions were correctly located, we performed angle comparison between the amino acids that coordinate copper ions for 1GYC and those found in the LaccGluc and LaccPost models generated (Table 1).

**Table 1:** Angle formation between amino acid atoms and copper ions in template and generated models.

**Active site**	***T. versicolor***		***G. lucidum***		***P. ostreatus***	
	**Angle formation between amino acid and copper ion**	**Angle (°)**	**Angle formation between amino acid and copper ion**	**Angle (°)**	**Angle formation between amino acid and copper ion**	**Angle (°)**
T1	His458 (ND1)→Cu1→Cys453 (SG)	129.000	His460 (ND1)→Cu1→Cys455	129.007	His458 (ND1)→Cu1→Cys453	128.677
	His395 (ND1)→Cu1→Cys453 (SG)	126.160	His397 (ND1)→Cu1→Cys455 (SG)	126.140	His396 (ND1)→Cu1→Cys453 (SG)	126.462
	His458 (ND1)→Cu1→His395 (ND1)	104.438	His460 (ND1)→Cu1→His397 (ND1)	104.447	His458 (ND1)→Cu1→His396 (ND1)	104.432
T2	His398 (NE2)→Cu4→His64 (NE2)	174.324	His400 (NE2)→Cu4→His66 (NE2)	174.306	His399 (NE2)→Cu4→His66 (NE2)	174.794
T3	His111 (NE2)→Cu2→His400 (NE2)	101.998	His113 (NE2)→Cu2→His402 (NE2)	102.002	His113 (NE2)→Cu2→His401 (NE2)	101.330
	His452 (NE2)→Cu2→His400 (NE2)	101.268	His454 (NE2)→Cu2→His402 (NE2)	101.295	His452 (NE2)→Cu2→His401 (NE2)	100.694
	His111 (NE2)→Cu2→His452 (NE2)	106.002	His113 (NE2)→Cu2→His454 (NE2)	106.009	His113 (NE2)→Cu2→His452 (NE2)	106.416
	His454 (NE2)→Cu3→His66 (ND1)	107.135	His456 (NE2)→Cu3→His68 (ND1)	107.114	His454 (NE2)→Cu3→His68 (ND1)	107.347
	His109 (NE2)→Cu3→His66 (ND1)	125.396	His111 (NE2)→Cu3→His68 (ND1)	125.392	His111 (NE2)→Cu3→His68 (ND1)	125.268
	His454 (NE2)→Cu3→His109 (NE2)	111.511	His456 (NE2)→Cu3→His111 (NE2)	111.540	His454 (NE2)→Cu3→His111 (NE2)	112.384

### LaccGluc and LaccPost validation models

**Figure 3:**
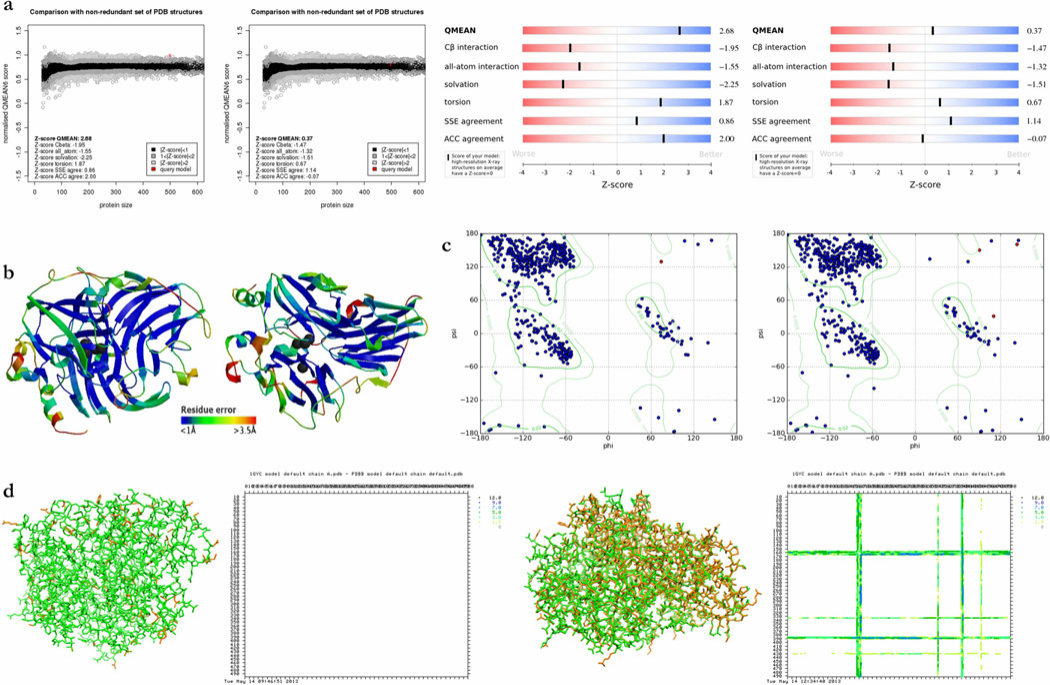
Model validation. For all graphs from left to right, first for LaccGluc followed by LaccPost. **a.** QMEAN distribution and bar graphs. Distribution graphs illustrate QMEAN score tendencies from high-resolution PDB crystallographic structures. Bar graphs help identify “acceptable” or positive scores (blue) from “unacceptable” or negative scores (reed) for each description. **b.** 3D structure with color gradient from blue to red defining regions with resolutions between 1 and 3.5 Å respectively. The greater blue values represent a more reliable structure. **c.** Ramachandran plots. We inferred model residues make part of α-helices and β-sheet and beta turns since they occupy permitted spaces. Red points correspond to amino acids different than glycine and proline. **d.** Ball-and-stick model between the model and its template, *DD* matrices estimated by SuperPose. *G. lucidum* and *P. ostreatus* models in orange, 1GYC structure in green. Overlapping was visualized by Chimera. *DD* matrices demonstrated the comparison between model principal chains (x-axis) and the template (y-axis). Differences between 0 and 1.5 Å in white, between 1.5 and 3 Å in yellow, between 3 and 5 Å in light green, between 5 and 7 Å in turquoise, between 7 and 9 Å in dark blue and over 9 Å in black i.e. the darker the color the greater the distance difference between structures.

Values obtained for each structural QMEAN described for each model are summarized in table 2. With respect to secondary structure (Q3 index) we obtained 83.5% for LaccGluc and 84.9% for LaccPost. LaccGluc and LacPost protein models had QMEAN scores of 0.988 and 0.797 respectively (Fig. 3a).

**Table 2:** QMEAN model results predicted by homology.

**Descriptor**	***G. lucidum***		***P. ostreatus***	
	**Valor**	**Score-Z**	**Valor**	**Score-Z**
Cβ energy of interaction	-33.21	-1.95	-73.30	-1.47
Interaction energy among all atoms	-8873.68	-1.55	-9737.41	-1.32
Solvation energy	-15.29	-2.25	-25.32	-1.51
Torsion angle energy	-189.62	1.87	-155.75	0.67
Secondary structure	83.5%	0.86	84.9%	1.14
Solvent accesibility	91.6%	2.00	80.8%	-0.07
QMEAN score	0.988	2.68	0.797	0.37

We describe six results obtained by QMEAN description. First four are energy values in kJ mol^-1^. Fifth is the secondary structure Q3 index. Last, is accessibility percentage, describing to what extent a structure is exposed. Each description has its Score-Z.

Ramachandran plots generated by UCSF Chimera demonstrated most residues for both structures (92% for LaccGluc and 91% for LaccPost models) occupied permitted regions (Fig. 3c). Some residues outside the allowed regions corresponded to glycine and proline. However, Leu60 in the LaccGluc model, and His166, Leu339, and Thr434 in the LaccPost model were distributed in regions that are not permitted (2% average for both models).

SuperPose analysis revealed the LaccGluc model was similar to 1GYC laccase in *T. versicolor*. In addition, backbone RMSD values and all atoms of specific segments for both sequences were 0.0 Å and 0.33 Å respectively. Small changes in some lateral LaccGluc chains were different compared to 1GYC resulting in a change for total atom RMSD values (0.33 Å). With regards to overlapping between LaccPost and 1GYC, some regions demonstrated significant differences (over 3 Å), presenting a backbone RMSD of 1.23 Å, and a 1.35 Å RMSD for all atoms in specific regions for both sequences (Table 3).

**Table 3:** VADAR prediction Model molecular characteristics.

**Characteristic**	***G. lucidum***	***P. ostreatus***	**Expected value (EV)**
α-helix	31 (6%)	31 (6%)	-
β-sheet	242 (48%)	238 (47%)	-
Coil	225 (45%)	227 (45%)	-
Turn	148 (29%)	136 (27%)	-
Hydrogen bond average distance (Å)	2.1 (ds 0.3)	2.1 (ds 0.3)	2.2 (ds 0.4)
Hydrogen bond average energy (kJ mol^-1^)	-2.0 (ds 0.9)	-2.0 (ds 0.9)	-2.0 (ds 0.8)
Number of residues with hydrogen bonds	328 (65%)	323 (65%)	75%
Helix average (Φ)	-65.6° (ds 8.8)	-65.6° (ds 8.8)	-65.3° (ds 11.9)
Helix average (ψ)	-33.3° (ds 13.8)	-33.3° (ds 13.8)	-39.4° (ds 25.5)
ω average angle (>90°)	179.6° (ds 2.9)	179.6° (ds 2.9)	180° (ds 5.8)
Number of residues with ω angles <90°	5 (1%)	4 (0%)	-
Total ASA (Å^2^)	18450.8	19202.8	17735.3
Molecular weight (kDa)	53.84604	54.18090	-

Additionally, NetNGlyc program predicted six potential N-glycosylation sites: (residues 30, 41, 49, 274, 335, and 418) for LaccGluc and one position (residue 436) for LaccPost.

### Computational laccase active site modeling—Ligand molecular docking

**Figure 4:**
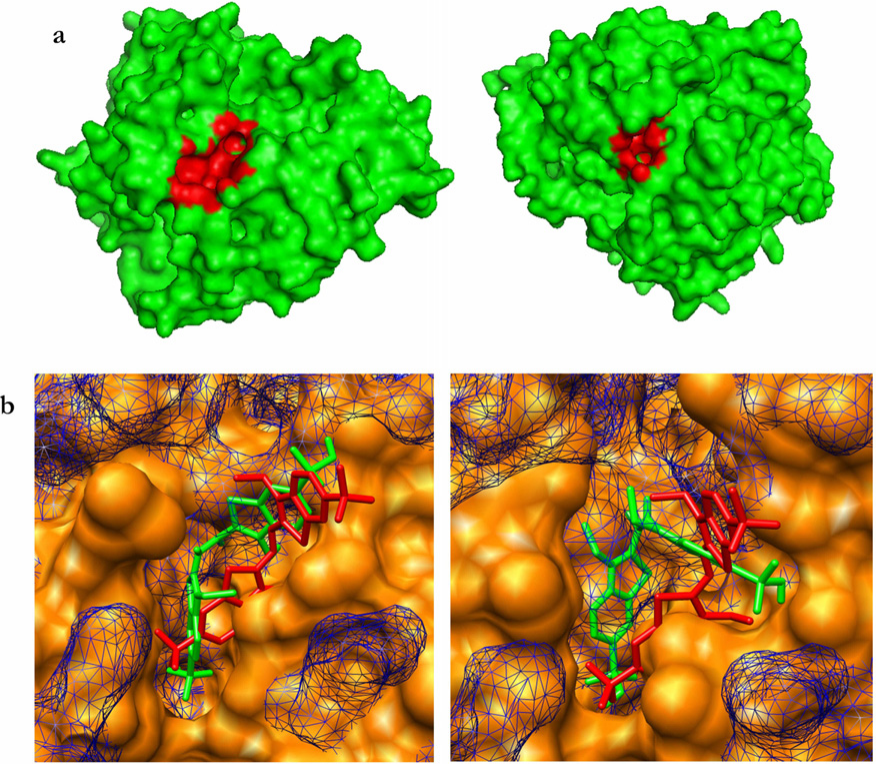
Active sites pockets models. **a.** LaccGluc and LaccPost T1 active site pocket surface. **b.** Comparison between the best pose with co-crystalized 3ZDW structure. Solid LaccGluc and LaccPost surfaces are in orange. 3ZDW: Gridded blue surface. Ligand in docking complex is depicted in green, and 3ZDW in red. All images were obtained from Chimera.

CASTp estimated for LaccGluc on laccase surface 84 pockets. One hundred pockets were estimated for LaccPost. We elected the nearest pocket to the T1 active site for each molecule (Fig. 4a), according to the pBLAST results. For LaccGluc the pocket was made of 20 different amino acids (Phe164, Pro165, Leu166, Asp208, Pro209, Asn210, Phe241, Ser242, Ser266, Phe267, Phe334, Phe339, Pro393, Gly394, Ala395, Pro396, Pro398, Ile457, Phe459 y His460). This pocket had an area of 133.032 Å^2^. For LaccPost it was constituted by 18 amino acids (Tyr154, Pro165, His166, Pro167, Asp207, Ser208, Asp209, Phe240, Ala241, Asp265, Ser266, Phe392, Ala393, Gly394, Pro395, Pro397, Ile455 y Trp457). This pocket had a slightly greater area of 133.779 Å^2^. Amino acid clusters that made-up the pockets were principally hydrophobic.

Out of the ten outputs from each ABTS pocket conformation simulation, none was below the cutoff point (2 Å). Nonetheless, selected RMSD values were closest to 3ZDW from *Bacilus subtilis* laccase co-crystalized with ABTS in its structure. For the LaccGluc complex we chose 4.036 Å for the RMSD value, with bond energy of -4.97 kcal mol^-1^, obtained from a 50^3^ grid. The LaccPost attained the best RMSD value (5.618 Å) with a 40^3^ grid, and a bonding energy of -4.15 kcal mol^-1^. Surface comparison between co-crystalized structure and docking complex (Fig. 4b), demonstrated a clear pocket difference. Divergence between model structure atomic distances and 3ZDW generated a distinct laccase surface topology pattern.

**Figure 5:**
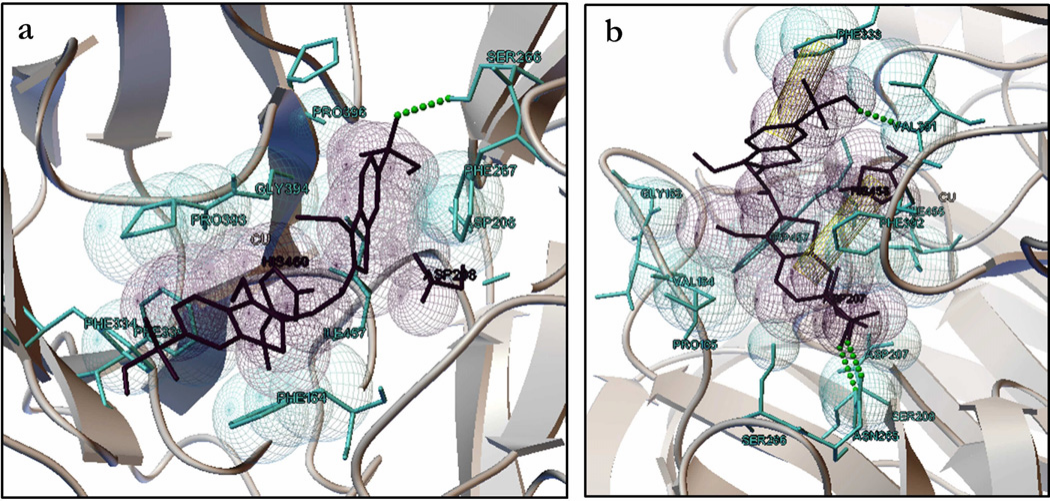
LaccGluc and LaccPost ABTS model interaction. **a.** LaccGluc **b.** LaccPost. Van der Waals residue-ABTS radii. Blue stick models: residue lateral chain. Purple stick models: ABTS and residues with flexibility. Yellow cylinders: π-π interactions, green spheres: hydrogen bonds. Models were visualized in AutoDock.

Laccase LaccGluc ABTS and model interaction were generated with the following amino acids: Phe164, Asp206, Ser266, Phe267, Asp298, Phe334, Phe339, Pro393, Gly394, Pro396, Ile457 and His460. For LaccPost with: Gly163, Val164, Pro165, Asp207, Ser208, Asn265, Ser266, Val391, Phe333, Phe392, Ile455, Trp457 and His458 (Fig. 5).

### pGAPZαA-*LaccGluc-Stop* and pGAPZαA-*LaccPost-Stop* vector construction, laccase LaccGluc and LaccPost expression clone screening

Results for pGAPZαA-*LaccGluc-Stop* and pGAPZαA-*LaccPost-Stop* vectors are summarized in Fig. 1b,c. Synthetic sequences obtained after DNA optimization with 5’ restriction site addition for *Eco*RI and *Not*I for the 3’ end are shown in Fig. 1a. Final LaccGluc synthetic gene construct size was 4605 bp and 4646 bp for LaccPost.

After electroporation of *P. pastoris* X-33 with synthetic vector, various clones were obtained demonstrating enzymatic activity on YPD agar dish supplemented with ABTS and CuSO_4_. Depending on ABTS oxidative state a green or purple halo was observed around the colonies, revealing recombinant enzyme production actively secreted into the media. Two X-33/pGAPZαA-*LaccGluc-Stop* and three X-33/pGAPZαA-*LaccPost-Stop* transformants with the greatest coloration were selected for recombinant enzyme production.

### Laccase LaccGluc and LaccPost constitutive expression preliminary studies

**Figure 6:**
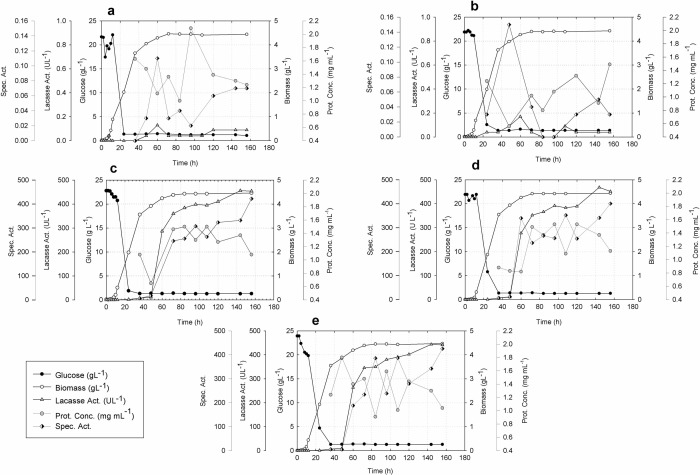
Laccase producing clones: growth, residual glucose, biomass and laccase enzyme specific activity. **a.** Clone 1 X-33/pGAPZαA-*LaccGluc-Stop*. **b.** Clone 2 X-33/pGAPZαA-*LaccGluc-Stop*. **c.** Clone 1 X-33/pGAPZαA-*LaccPost-Stop*. **d.** Clone 2 X-33/pGAPZαA-*LaccPost-Stop*. **e.** Clone 3 X-33/pGAPZαA-*LaccPost-Stop*. Clones containing the gene from *G. lucidum* (**a, b**) had a specific activity of the enzyme considerably lower than for clones containing the gene from *P. ostreatus* (**c, d, e**). It also notes that *G. lucidum* clones generated under this assays conditions the highest specific activity at the end of the exponential growth phase unlike the clones with the gene from *P. ostreatus* that showed the maximal specific activity at 156 hours of culture. However, in all cases it is observed that the trend in the cellular growth was very similar and that at the end of the exponential growth phase is achieved detecting specific activity.

Five clones were selected after *P. pastoris* X-33 transformation. Clone kinetic behavior at a 100 mL scale is illustrated in Fig. 6 and table 4.

**Table 4:** Recombinant laccase X-33/pGAPZαA-*LaccGluc-Stop* and X-33/pGAPZαA-*LaccPost-Stop* kinetic parameters.

**Constructs and kinetic parameters**	**Clone number**		
**X-33/pGAPZαA-*LaccGluc-Stop***	**Clone 1**	**Clone 2**	
μ_(x)_ (h^-1^)	0.35	0.40	
t_d_ (h)	1.96	1.73	
P_(x)_ (gL^-1^ h^-1^), (Time of calculation h)	0.10 (36)	0.10 (36)	
P_(Enzyme)_ (UL^-1^ h^-1^), (Time of calculation h)	0.005 (8)	0.0028 (60)	
Specific Activity, (Time of calculation h)	0.11 (60)	0.15 (48)	
**X-33/pGAPZαA-*LaccPost-Stop***	**Clone 1**	**Clone 2**	**Clone 3**
μ_(x)_ (h^-1^)	0.40	0.54	0.42
t_d_ (h)	1.74	1.29	1.63
P_(x)_ (gL^-1^ h^-1^), (Time of calculation h)	0.10 (36)	0.10 (36)	0.10 (36)
P_(Enzyme)_ (UL^-1^ h^-1^), (Time of calculation h)	5.00 (72)	4.89 (72)	4.80 (72)
Specific Activity, (Time of calculation h)	422.20 (156)	400.00 (156)	425.69 (156)

Parameters were calculated from clones producing recombinant laccase X-33/pGAPZαA-*LaccGluc-Stop* and X-33/pGAPZαA-*LaccPost-Stop*.

μ_(x)_ (h^-1^): specific growth rate; t_d_ (h): duplication time; P_(x)_ (gL^-1^ h^-1^): biomass productivity; P_(Enzyme)_ (UL^-1^ h^-1^): enzyme productivity

## Discussion

### LaccGlucc-Stop and LaccPost-Stop design and optimization

Unlike many constructs designed for *P. pastoris’* recombinant protein expression, ours used *P. pastoris* X-33 GAP constitutive promoter. In this way assured the recombinant protein would be expressed in a constitutive manner, based on glucose addition as the carbon source. We designed our constructs to be used for environmental applications because our design avoids methanol addition as an *AOX*1 promoter inducing agent [26]; our system could be considered more eco-friendly to the environment in comparison to other laccase constructs [15, 16, 26].

In addition, to facilitate protein secretion to the culture media we selected *P. pastoris’ α-factor* putative leader sequence from *S. cerevisiae*. Therefore, we eliminated nucleotide sequences corresponding to native signaling peptides: residues 1–63 in *GlLCC1* and 1–60 in *POXA 1B*. Protein production was evidenced by ABTS and CuSO_4_ addition, based on green or purple halo intensity around the colonies.

In our design we maintained stop codons to guarantee translation termination at the end of each gene. In addition, our designed pGAPZαA vector does not translate *myc* epitope sites and the 6 Histidine tail present downstream the multicloning site. Thus, by using synthetic genes for optimized laccases in *P. pastoris*, GC content, *Cis* regulatory elements, repeated sequences, and signaling peptide we expected to facilitate heterologous protein production secreted to the culture media.

### Structural analysis in models constructed by homology

An important issue in homologous model is selecting an appropriate template for the protein of interest model construction. Until now various fungal laccases and bacteria structures have been determined by crystallography [9]. The models proposed in this work are sufficiently accurate that exceed the minimum identity percentage for models constructed by modelling [27]. In addition, they exhibited a molecular architecture previously described for other laccases, where three cupredoxin domains are clearly identified (Fig. 2), naturally occurring in laccase proteins [9]. 3DLigandSite prediction and overlapping between the models and the template were sufficiently precise to model missing copper ions and even oxygen atoms coordinating with these ions (Fig. 2).

### Model validation

3D model validation is an essential step that can be performed at different levels of structural organization to evaluate stereochemical parameters and folding precision. Energy values obtained by QMEAN for the LaccPost model are lower compared to LaccGluc, with the exception of torsion angle energy, which was higher. Highly confident values are considered when the Q3 index is above 75% [28], suggesting the models present an accurate secondary structure. Lastly, structure solvent accessibility was present, since accessibility values were higher that 25% [29].

Consensus values for each descriptor were represented by QMEAN score, indicating 3D global structure for each model was similar to native forms, since this is the structure distribution range for non-redundant crystallographic structures (Table 2, Fig. 3a).

For the Ramachandran plots we observed glycine permitted conformational flexibility, because as a residue it has hydrogen in its side chain. Due to the simplicity of its structure it allows to form turns that can be critical in comparison other amino acids. On the contrary, proline has a limited number of possible conformations. Proline’s aliphatic side chain is a cyclic structure. Since its nitrogen N-C _α_makes part of a rigid ring it reduces the structural flexibility of polypeptide regions containing proline. Thus it is common to find these residues outside of permitted regions in this type of analysis. Nonetheless, taking into account these residues are located in coil regions, it is common to find atypical flexibilities at these sites [30].

SuperPose analysis revealed LaccPost had less similarities compared to the template, probably due to the 60.8% identity present between superimposed structures (Fig. 3d). However, in general terms, model architecture present similar values to those previously reported for other laccases [31]. In contrast, the models presented high ASA values in comparison to VE values, demonstrating cavities and pockets in various regions of the protein, characteristic of enzymatic reactions [32], (Table 3).

By using NetNGly we found in *G. lucidum* laccase a carbohydrate content of 7% to 10% [33] and in *P. ostreatus* laccase 3% to 9% laccase content [34]. In addition to these percentages, VADAR estimated weighs for both structures were within the characteristic laccase weigh (50–60 kDa) [35].

### Ligand active site molecular docking of the modeled laccases

According to Cambria *et al.* (2010) physico-chemical characteristics found in LaccGluc and LaccPost pockets are characteristic of catalytic pockets. In laccases they are essential in preventing metallic ion contact with water, and facilitate hydrophobic substrate interaction with the enzyme’s active center. This cavity is formed by an α-helix and a β-sheet in the D3 domain (Fig. 3); commonly found in laccases [25, 36, 37].

When comparing co-crystallized structures with ABTS (3ZDW) and docking complexes (LaccGluc-ABTS and LaccPost-ABTS) (Fig. 4b), we found some differences, probably leading to steric hindrance by some residues that make-up the pockets in our models. This makes it difficult for ABTS to adopt closer configurations to those present in the 3ZDW complex (Fig. 4b). The greatest energy required to generate an ABTS inter-atomic interaction with LaccPost could be explained by irregularities within the pocket with respect to the LaccGluc model.

It is noteworthy to mention values closer to the cutting point can be obtained by increasing the AutoDock output. Previous studies have reported RMSD values close to a 2.2 Å between *T. versicolor* and *B. subtilis* laccase complex [38]. These values could be achieved by using other programs such as Gold [39] by authors. However, it is possible to obtain values close to 2.2 Å in the present study (using AutoDock), due to the nature of the pocket’s topological structure similar to previously reported and to the “very elevated” bond energies, compared with other models that were found.

Despite a similar physico-chemical residue arrangement our models presented a greater number of interactions with respect to the number of residue interactions in the 3ZDW-ABTS complex (Pro226, Ala227, His319, Cys322, Gly323, His497, Pro384, Arg416 y Gly417). It is important to highlight that *B. subtilis* laccase’s side chain and the ABTS interaction is fundamentally generated with a histidine residue (His497) coordinating a T1 copper. Enguita *et al.* (2004) proposed that working under low resolution (2.4 Å) it is not possible to observe the type of interaction produced between the residue and ABTS. However, for our docking evaluations residues equivalent to 3ZDW histidine generated Van der Waals interactions with ABTS (His460 and His458 for LaccGluc and LaccPost models). Furthermore, for the LaccPost model a π-π interaction was generated between the His458 imidazol ring and ABTS’ benzene ring. Importance of this histidine ring coordinating T1 copper is due to its function as a “gate” coordinating electronic transfer [40]. This is a critical step to initiate substrate degradation in laccase’s catalytic cycle.

For 3ZDW crystallographic structure we found a pattern for ABTS. The dye is partially embedded by laccase and one of the thiazoline rings is perpendicular to the His497 imidazol group. Furthermore, thaizoline rings are in a *trans* position, favoring electronic transfer [41]. For the LaccGluc-ABTS and LaccPost-ABTS docking complexes we observed a perpendicular pattern, with *cis* isometry (Fig. 5). Apparently, in the models ABTS cannot adopt a *trans* position, due to steric hindrance generated by some residues that make-up the pockets (Fig. 4b).

For *B. subtilis* laccase a disulphide bond is generated between Cys229 and Cys322, where one of the ABTS sulphonate group tends to make contact with the disulphide bond. In addition, an oxygen atom in the sulphonate forms a hydrogen bond with Gly323 [41]. For the LaccGluc-ABTS and LaccPost-ABTS models, sulphonate group contact is not feasible due to the distance between disulphide and T1. A hydrogen bond is produced between sulphonate oxygen and other residues other than glycine: Ser266 for LaccGluc. For LaccPost the residues were Ser208, Asn265 and Val391. Even though our results do not agree with those reported by Enguita *et al.* (2004), it is important to highlight laccase’s interactions are different among bacterial and fungal species, since they present different sizes and conformations. Prasad *et al.* (2012) reported for *P. cinabarinus* laccase hydrogen bond interaction between its asparagine and the oxygen in ABTS sulphonate, and thiazoline *cis* ring configuration. These configurations are similar to those observed in the LaccPost-ABTS complex (Fig. 5).

### pGAPZαA-*LaccGluc-Stop* and pGAPZαA-*LaccPost-Stop* vector construction, laccase LaccGluc and LaccPost clone screening

Obtained constructs by restriction enzyme analysis demonstrated they were in frame with the transcription direction and downstream the signal peptide. Synthetic genes coding for LaccGluc and LaccPost enzymes contained *Eco*RI and *Not*I restriction enzyme sites at the 5´ and 3´ ends generated by us (Fig. 1b, c).

### Laccase LaccGluc and LaccPost constitutive expression preliminary studies

Laccases LaccGluc and LaccPost synthetic genes were expressed in *P. pastoris* under glyceraldehyde-3-phosphate dehydrogenase (GAP) constitutive control and both recombinant enzymes were secreted to the culture because of *P. pastoris α*-factor leader.

Fig. 6 illustrates kinetic behavior for all five recombinant clones containing synthetic genes from *G. lucidum* laccase (*LaccGluc-Stop*). For *P. ostreatus* (*LaccPost-Stop*) they were similar. A maximum biomass average production of 4.45 ± 0.017 gL^-1^ at 156 h of culture was obtained. Nonetheless, laccase enzymatic activity was only relevant for three clones with the synthetic gene from *P. ostreatus* (LaccPost) laccase. An average laccase activity of 451.08 ± 6.46 UL^-1^ between 144 h and 156 h of culture was obtained. Likewise, specific enzyme activity for these three clones was produced after 156 h of culture with an average extracellular protein value of 416.14 ± 13.64 UL^-1^ mg^-1^. Enzymatic activity for the two clones with the synthetic gene from *G. lucidum* (LaccGluc) did not exceed 0.2 UL^-1^ (Fig. 6).

Kinetic parameters calculated from preliminary assays are shown in table 4. They corroborate a similar tendency among the five clones with respect to growth, and enzyme activity discrepancies.

We observed enzyme activity differences between clones X-33/pGAPZαA-*LaccPost-Stop* and X-33/pGAPZαA-*LaccGluc-Stop*. We suggest the following possible scenarios. First, these discrepancies could be related to catalytic pocket structural and topological characteristics for both enzymes. Van der Waals and π-π interactions formed in the LaccPost-ABTS complex lead to a greater substrate affinity, in contrast to what was observed for LaccGluc-ABTS (Figs. 4 and 5). Thus, a greater activity was observed for the LaccPost clones.

A second plausible explanation could be related to the number of synthetic gene copies. It is possible that during transformation a greater number of *LaccPost-Stop* copies remained in the yeast chromosome; which has to be shown in our case.

Generally, laccase activity is favored in a certain way by copper concentration [9, 19, 42]. Based on literature [19], we used the lower CuSO_4_ concentration (0.1 mM CuSO_4_) for our assays, which to date have not been optimized. Another possible justification for enzyme activity differences could be CuSO_4_ concentration, favorable for *LaccPost-Stop* clones and maybe to less extent for *LaccGluc-Stop*.

In this work we only estimated enzyme activity, without evaluating specific enzyme concentration by an ELISA identifying inactive enzyme. In addition, we have not quantified mRNA by qRT PCR determining gene expression levels. Both aspects could be influencing gene expression, thus explaining divergence between gene expression and specific enzyme activity. We have begun to characterize gene expression levels through qRT PCR for both genes to associate them to produced enzyme and biological activity.

All these aspects and other, which we have not considered, could be influencing individually or collectively gene expression, protein synthesis, and enzyme activity for each of the clones.

Last, we demonstrated in this work computational *GlLCC1* from *G. lucidum* and *POXA 1B* from *P. ostreatus* optimization was beneficial to achieve constitutive expression under GAP promoter and α-factor in *P. pastoris*. We proposed and validated a 3D computational model for each enzyme and observed the LaccGluc proposed model was similar to *T. versicolor* 1GYC 3D crystallographic structure. After analyzing with molecular docking ABTS interaction between both models, we evidenced for each enzyme studied ABTS substrate-enzyme interaction occurred at the enzyme’s active site. Moreover, we found LaccPost had a greater affinity for ABTS than LaccGluc. This finding was validated by our experimental approach through the kinetic preliminary study. Under the same assay conditions with same culture media, laccase activity in UL^-1^ and specific activity in UL^-1^mg^-1^ extracellular protein was greater for the three *LaccPost-Stop* synthetic genes from *P. ostreatus* clones. In contrast, *LaccGluc-Stop* from *G. lucidum* had lower values for specific activity and extracellular protein. Optimization studies for media and culture conditions should have a positive effect on the clone’s kinetic behavior and enzyme biological activity for both recombinant laccases; work that is presently in progress.

Our findings are important in light of recombinant enzyme expression system utility for environmentally friendly designed expression systems. Laccases from white-rot fungi Basidiomycetes have great biotechnological potential due to the wide range of substrates they can transform. These include phenolic compounds, which are difficult to degrade using redox mediators, of natural or synthetic origin. In addition, high redox laccase potential can be used in almost all manufacturing paper products: pulp production, pulp chlorine-free bleaching, or effluent treatment.

In the forest industry laccases can be used for lignocellulosic material design with novel properties, such as stability resistance by grafting laccase catalyzed phenolic compounds. This treatment can improve wood board adhesion by enzymatic “*in situ*” lignin coupling, without using toxic formaldehyde based adhesives. In addition, laccases play an important role in other fields such as the food processing industry in beverage or bakery products. Moreover, it is employed in the textile industry in dye effluent detoxification or fabric bleaching. Furthermore, in nanobiotechnology it has been used in the development of biosensors for clinical and environmental analysis such as phenol, oxygen, azide, morphine, codeine, or flavonoid catecholamines detectors. Moreover, it has applications in fuel biocell development, offering clean electricity without fossil fuel use, by immobilizing laccases at the cathode. Lacasses can degrade polyethylene, bioremediate PAHs, synthesize complex polymers such as polycatechol chromatography resins, act as an antitumor agent, antibiotic, and formulated hair tempt laccase [9, 43].

Conclusively, white rot fungi laccase recombinant products contribute to a wide gamut of products in diverse settings: industry, clinical and chemical use, and environmental applications.

## Materials and Methods

### *P. ostreatus* and *G. lucidum* laccases synthetic sequence design and optimization

*P. ostreatus* Lacc6 or POXA 1B was designed based on a *Joint Genome Institute*—USA sequence (http://genome.jgi.doe.gov/PleosPC15_2/PleosPC15_2.home.html) [44]. *G. lucidum,* GlLCC1 laccase was designed from a GenBank sequence access number FJ656307.1 [16, 45]. Both sequences were optimized using OptimumGene (GenScrip, Piscataway, NJ USA). Optimized genes were named *LaccGluc-Stop* (*G. lucidum* laccase with a stop codon) and *LaccPost-Stop* (*P. ostreatus* laccase with a stop codon). This optimization was performed to adjust codon use, GC content, and *Cis* regulatory elements, and repetitive sequence presence for their expression in *P. pastoris*. Additionally, the native signal peptide was removed and restriction enzyme sites for *Eco*RI at 5´ and *Not*I t 3´ were added, to facilitate expression vector construction. Optimized sequences were synthesized by Gen Script.

### 3D models

Protein sequences and their respective models obtained by conceptual translation from optimized laccase genes in *G. lucidum* GlLCC1 and *P. ostreatus* POXA 1B were named *LaccGluc-Stop* and *LaccPost-Stop* respectively. Sequences were uploaded on line to the *Phyre 2.0* server (*Structural Bioinformatics Group* Imperial College, London UK) [46]. Phyre identified for both proteins *T. versicolor* laccase crystalographic structure registered in PDB with access code 1GYC. Phyre is associated with the 3DLigandSite server (*Structural Bioinformatics Group* Imperial College, London UK) capable of generating possible ligand clusters for certain protein active sites [47]. Using Pfam data base [48, 49] different structural domains were identified using Chimera software (University of California San Francisco, USA) [50]. Computatinal models were overlapped with 1GYC structure to eliminate errounseously estimated copper ions, leaving only those ions that better adjusted to the template. Estimation only ocured for T2/T3 site. Therefore, based on the overlapping results to add missing ions, we used T1 copper coordinate sites that were later added to the coordinate file for each model modifying the HETAM function.

### Model analysis and evaluation

Geometry analysis was performed by the QMEAN server *Swiss Institute of Bioinformatics* (SIB). QMEAN estimates the modeled structure’s quality conducting an exhaustive comparison with PDB structures determined by crystallography or magnetic resonance. Scores are given by two scoring functions QMEANlocal y QMEANclust [51–53]. To estimate correct dihedral angle (ψ/Ф) disposition a Ramachandran plot was generated ignoring glycine and proline residues by means of the Chimera program. RMSD analysis was achieved by comparing each model with *T. versicolor* structure using the SuperPose 1.0 program [53, 54]. Model architecture was analyzed by VADAR 1.8, which calculates average hydrogen bond distance, dihedral angles, accessible surface area, and volume [32].

### Ligand and molecular docking models

We selected ABTS as the ligand model from NCBI’s Pubchem Simplified Molecular Line Entry Specification (SMILES) [54]. Structures were modeled and refined in Chimera. Before performing docking simulations with Autodock 4.2 [55], laccase surface substrate accessibility and catalytic site residue implications were identified by CASTp [56]. Polar hydrogens were added to each receptor, and charges were included by the Gasteiger methods to each receptor and ligand [57]. To include copper atom charges, AutoDock’s receptor preparation coordinate file was modified. According to CASTp results the grid was localized in the active site pocket for each model. Two different dimensions (40^3^ and 50^3^ points) were used for ABTS box determination with a 0.375 Å space. Grid parameters and atomic affinity maps were calculated with AutoGrid 4. Each docking simulation was carried out with Lamarkian genetic algorithm with 2,500,000 energetic evaluations with a population of 150. In order to elect the ABTS configuration closest to a crystallographic complex, predicted couplings were compared with 3ZDW laccase co-crystalized with ABTS, measuring RMSD for each estimate by using Chimera. Last, ligand-model molecular interactions were determined with bond energy results are given in kcal mol^-1^.

### Plasmid and media *LaccGluc-Stop* and *LaccPost-Stop* synthetic gene cloning and expression

DH5*α Escherichia coli* (F- *end*A1, *gln*V44, *thi*-1, *rec*A1, *rel*A1, *gyr*A96, *deo*R, *nup*G, Φ80Δ*lac*ZΔM15 Δ(*lac*ZYA-argF)U169, *hsd*R17(r_K_-m_K_+), λ–) was used as a host for subcloning assays. For recombinant laccase expression wild type*P. pastoris* X-33 was utilized as a host. pUC57 was selected to construct pUC57-*LaccGluc-Stop* and pUC57*-LaccPost-Stop* shuttle vectors. pGAPZαA was elected as a vector for constitutive expression. DH5*α E. coli* was cultured in Luria-Bertani (LB) media (1% w/v Tryptone, 0.5% (w/v) yeast extract, 0.5% (w/v) NaCl, pH 7.5 ± 0.2) or in LB agar Petri dishes (LB media with 1.5% (w/v) agar-agar). Ampicillin at 100 µg mL^-1^ or Zeocin at 40 µg mL^-1^ were used as selection markers. Additionally 0.2 mM ABTS and 0.1 mM CuSO_4_ were added.

### *P. ostreatus* and *G. lucidum* laccase expression vector construction

DNA manipulations and cloning were carried out using previously standardized procedures [58]. Once expanded, pUC57-*LaccGluc-Stop* and pUC57-*LaccPost-Stop* were digested simultaneously with *Eco*RI and *Not*I (Promega, Madison WI USA). Enzyme digestion products were evidenced in 1% TAE agarose gel, extracted and purified with *and PCR Clean-Up System* (Promega). Purified fragments were ligated using *LigaFast* (Promega) to constitutive expression vector pGAPZαA that was previously digested with *Eco*RI and *Not*I (Promega) resistant to Zeocin^TM^. DH5*α E. coli* was transformed with ligation products, and selected for their capacity to grown in LB media supplemented with Zeocin at 40 µg mL^-1^. Plasmid DNA extraction was carried out by using *Miniprep Purification System* (Promega). Expression vectors were identified as pGAPZαA-*LaccGluc-Stop* and pGAPZαA-*LaccPost-Stop*. To demonstrate insert presence construct pGAPZαA-LaccGluc-Stop was digested with *EcoR*I and *BamH*I. pGAPZαA-LaccPost-Stop was digested with *EcoR*I, *BamH*I, and *Hind*III. All restriction enzymes were purchased from New England BioLabs (New England BioLabs, Ipswich MA USA).

### *P. pastoris* expression strain screening

pGAPZαA-*LaccGluc-Stop* and pGAPZαA-*LaccPost-Stop* expression vectors used in the transformation were linearized with *Avr*II (New England BioLabs, Ipswich MA USA). Competent *P. pastoris* X-33 cells were transformed by electrophoresis with each vector respectively following the manufacturer’s instructions (Invitrogen, Carlsbad CA USA). Approximately 1 µg of linearized vector was used for 80 µL competent *P. pastoris* X-33 solution. Immediately after a 1.5 kv pulse for 5 ms, 1 mL of 1 M sorbitol at 0°C was added to the electroporation cuvette.

After 2h incubation at 30°C without shaking, 1 mL YPD media was added and further incubated for 3h at 30°C and 200 rpm. Last, media was seeded onto Petri dishes containing YPD supplemented with Zeocin 100 µg mL^-1^, 0.2 mM ABTS, and 0.1 mM CuSO_4_. Petri dishes were incubated at 30°C and observed on a daily basis until colonies appeared. Depending on ABTS’ oxidation state a green or purple halo was formed. This halo resulted from recombinant laccase action on ABTS substrate. Control colonies did not produce any halo. Clones obtained by this assay were named X-33/pGAPZαA-*LaccGluc-Stop*, X-33/pGAPZαA-*LaccPost-Stop* and X-33/pGAPZαA.

### Enzyme assays

Laccase enzyme activity was monitored by a change in absorbance at 436 nm (ξ_436_=29300 M^-1^ cm^-1^) due to ABTS oxidation in a 60 mM sodium acetate buffer (pH 4.5 ± 0.2). 800 µL room temperature crude extract was added to 100 µL 600 mM sodium acetate buffer, and 100 µL 5 mM ABTS. Green radical formation was evaluated spectrofotometrically during 3 minutes. A unit of activity is defined as the quantity of enzyme required to permit oxidation of 1 µmol ABTS per minute. Negative control solution contained 800 µL de distilled water, 100 µL 600 mM sodium acetate buffer solution, and 100 µL 5 mM ABTS. Enzyme activity was expressed as UL^-1^ [59].

### *Pichia pastoris* expression preliminary studies

A colony of positive transformants for each case, pGAPZαA-*LaccGluc-Stop* or pGAPZαA-*LaccPost-Stop*, was cultured in 50 mL tubes at 30°C in 5 mL YPD media between 16 h to 20 h with 210 rpm shaking until an OD_λ600nm_ reading between 2–5 was attained. Two ml inoculum were cultured in 98 ml YPD in 500 mL Erlenmeyers (2% v/v). Cultures were monitored by OD (OD_λ600nm_), total extracellular proteins were determined by Biuret assay [60], residual glucose (g L^-1^) by 3,5-dinitrosalicilic acid (DNS) [61], and laccase activity (U L^-1^) using ABTS as a substrate [59]. Samples were collected every 2 h for the first 12 h, followed by collections every 12 h until 156 h of culture were completed. To calculate cellular biomass concentration a calibration curve (X _(g DCW L_^-1^_)_ vs. OD_λ600nm_) was employed represented in equation *I*.

$$X=1.1726\times OD_{\lambda 600nm}(R^{2}=0.987),$$ (1)

**Where:** *X* = gL^-1^ of Dry Cell Weight (DCW)

*X* (gL^-1^ of DCW) data was transformed as *Ln* (*x*/*x*_0_) and plotted vs. time (h). Specific growth rate *μ_(x)_* (h^-1^) was calculated using the exponential phase slope (Equations *II*—*IV*), and duplication time *t_d_* (h), (Equation *V*), [12, 62, 63].

$$\mu_{\left(x\right)}=\frac{1}{x}\frac{dx}{dt},$$ (2)

$$Lnx=Lnx_{0}+\mu_{\left(x\right)}t,$$ (3)

$$Ln\left(\frac{x}{x_{0}}\right)=\mu_{\left(x\right)}t,$$ (4)

**Where:** *t* = *t _log phase_-t _lag phase_*

$$t_{d}=\frac{Ln2}{\mu_{\left(x\right)}},$$ (5)

Specific activity was calculated by dividing enzymatic activity for each hour of culture by total extracellular protein concentration (Equation VI).

$$Act.Esp.=\frac{Act.Enz.}{Conc.\mathrm{Pr}ot},$$ (6)

**Where**: *Act.Enz* =U L^-1^, *Conc.Prot*. = mg mL^-1^

Biomass productivity in gL^-1^ h^-1^ (Equation VII) as a function of enzyme biological activity UL^-1^ h^-1^ (Equation VIII) was calculated as follows [12, 62, 63].

$$P_{\left(X\right)}=\frac{\left[DCW\right]}{t},$$ (7)

$$P_{\left(Enzyme\right)}=\frac{Act_{Enzyme}}{t},$$ (8)
